# Smoking Exposure and Survival of Patients with Esophagus Cancer: A Systematic Review and Meta-Analysis

**DOI:** 10.1155/2016/7682387

**Published:** 2016-03-17

**Authors:** Jun-jie Kuang, Zhi-min Jiang, Yan-xian Chen, Wei-peng Ye, Qiong Yang, Hui-zhong Wang, De-rong Xie

**Affiliations:** Department of Oncology, Sun Yat-sen Memorial Hospital, Sun Yat-sen University, Guangzhou, Guangdong 510120, China

## Abstract

Smoking is a well-known major risk factor in development of esophageal cancer, but few studies have reported the association between smoking status and prognosis of these patients. We conduct the present study to summarize current evidence. A computerized search of the PubMed and EMBASE was performed up to April 30, 2015. Eight studies, containing 4,286 patients, were analyzed. In the grouping analysis, among esophageal squamous-cell carcinoma patients, current and former smokers, compared to those who have never smoked, seemed to have a poorer prognosis (HR = 1.41, 95% CI 1.22–1.64, and HR = 1.35, 95% CI 0.92–1.97, resp.). In the subgroup analysis, adverse effects on current smoker compared with never smoker were also observed in China and the other countries (HR = 1.5, 95% CI 1.18–1.92, and HR = 1.36, 95% CI 1.12–1.65, resp.). In the group that ever smoked, we could not get a similar result. No significantly increased risk was found in esophageal adenocarcinoma patients compared to the squamous-cell histology ones. In the smoking intensity analysis, heavy smoking was associated with poor survival in esophageal squamous-cell carcinoma. Our pooled results supported the existence of harmful effects of smoking on survival after esophagus cancer diagnosis.

## 1. Introduction

 Esophageal cancer (EC) is the sixth leading cause of cancer mortality in the world. According to the official statistics of the US, about more than 18,000 cases were newly diagnosed and 15,000 deaths from EC in 2014, representing 5% of all cancer death [1]. Esophageal squamous-cell carcinoma (ESCC) and esophageal adenocarcinoma (EA) are the two main histological subtypes of esophagus cancer. Within recent decades, in some places, such as North America and Europe, the incidence and mortality rate of ESCC have decreased [2]. But in Asia, particularly in China, ESCC still occupies the vast majority of EC. Most patients with EC are already in locally advanced or metastatic disease at the time of diagnosis. With the lack of opportunity for radical surgery, radiation and chemotherapy became the major palliative treatment.

So far, sufficient population-based case-control and cohort studies have indicated that gastroesophageal reflux disease (GERD), cigarette smoking, and obesity are the main established risk factors for developing EC [3–8]. Studies have shown that, compared with nonsmokers, ESCC incidence risk is increased by approximately 3- to 7-fold in current smokers, and the risk of esophageal squamous-cell carcinoma is greater than adenocarcinoma [9]. But the relationship between smoking exposure and the prognosis of patients with esophagus cancer is still not clear. Thus, in this systematic review and meta-analysis, we perform a summary of literatures on the association between current, former, and never smoking behavior and survival of EC patients.

## 2. Materials and Methods

PubMed and EMBASE were searched to identify the related studies that had evaluated survival in association with smoking behavior in EC patients until 30 April, 2015. An effective search strategy was performed through keywords as follows: “smoking,” “cigarette smoking,” “tobacco smoking,” “esophageal cancer,” “esophageal squamous cell carcinoma,” “esophageal adenocarcinoma,” “oesophageal cancer,” “oesophageal tumor,” “gastro-esophageal junction cancer,” “prognosis,” and “survival”. In addition, the reference lists of relevant articles were manually searched to find any other potentially eligible articles.

### 2.1. Selection Criteria

Case-control studies or cohort studies about the relationship between smoking status and oncological prognosis of patients with EC or gastroesophageal junction cancer were eligible to be included. Articles about smoking intensity are also included in the present study. We excluded reviews, commentaries, articles from overlapping samples, conference abstracts, and articles printed in languages other than English. The impact of smoking on survival had to be quantified by effect index such as hazard ratios (HR) with 95% confidence intervals (CI). Quality of each eligible study was also rated independently by two reviewers. Finally, meeting abstracts were excluded. Two reviewers independently screened the database for titles and abstracts. If either reviewer though that certain title or abstract met eligibility criteria, the full text of the article was retrieved.

### 2.2. Data Extraction

Two reviewers (Jun-jie Kuang and Zhi-min Jiang) identified potentially relevant studies by screening titles and/or abstracts of all citations identified through the database search. The full manuscripts of all articles identified in the search were screened for eligibility criteria by two independent reviewers (Jun-jie Kuang and Zhi-min Jiang). Disagreements were resolved through discussion. When necessary, a senior professor (De-rong Xie) helped to reach a consensus with all investigators. When an article provided more than one estimate, we chose the one adjusted for the largest set of variables. If a study published several update results, only the latest one would be included and analyzed. According to Newcastle-Ottawa Scale (NOS), the quality of each included article was assessed. For each study, the following characteristics were extracted using a standardized form: first author, country, publication year, study design, collection time, follow-up time, cases and controls, gender of subjects, age, tumor stage, clear definition of smoking status, adjustment variables, histological type, HR, and 95% CI (Tables 1 and 2). In case of missing data, we contacted the primary investigators through emails.

### 2.3. Statistical Analysis

In the present meta-analysis, survival is measured from diagnosis to all-cause death. Those studies of low credibility, less than 6 points (NOS score range from 0 to 9 points), were excluded from the final meta-analysis [18]. We extracted HRs and estimated their standard errors indirectly with the help of Review Manage 5.3 software. Statistical heterogeneity was evaluated by the *Q* test [19]. For the *Q* statistic, a *p* value of less than 0.10 was considered statistically significant heterogeneity. If heterogeneity was detected (*p* < 0.10), random effects model was used; otherwise, fixed-effects model was used. Potential publication bias was identified by Begg's test and funnel plots [20]. Subgroup analysis based on geographical regions was also performed. All statistical analyses were carried out with Review Manage 5.3 version 5.3.3 (Cochrane Collaboration, Oxford, UK) and Stata version 12.0 (StataCorp, College Station, TX, USA) [21, 22].

## 3. Results

### 3.1. Study Characteristics

**Figure 1:**
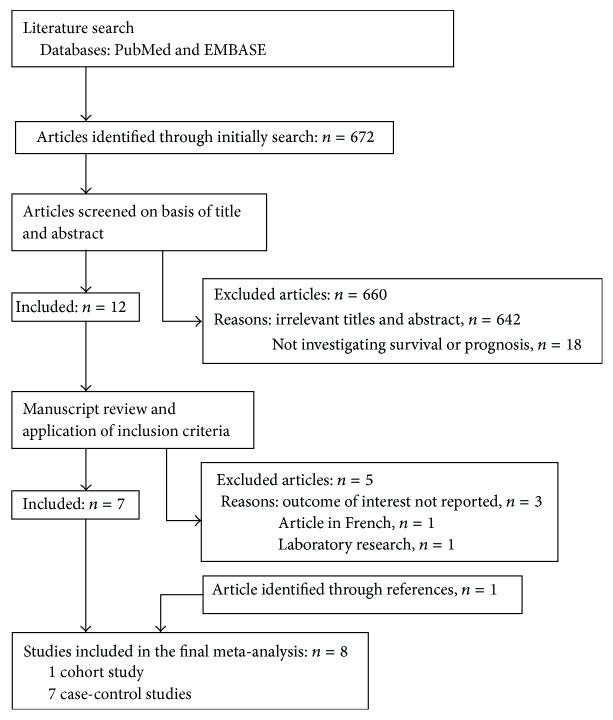
Flow chart of literature search for this meta-analysis.

A total of 672 articles were obtained through initial search. Of those, 660 were excluded by title and abstract scan and another five by full-text reading. One additional article was found in the references. Finally, eight studies [10–17] were eligible for a qualitative analysis, including one cohort study [14] and seven case-control studies [10–13, 15–17] (Figure 1). Reviewers had perfect agreement in selecting the eight studies using the stated eligibility criteria. The eight studies comprise data from a total of 4,286 cases that were published between 2005 and 2013. Three studies were carried out in China [10, 13, 14], and the other five studies were each from other countries (USA [11], Japan [12], Iran [15], Australia [16], and Sweden [17]). Sample size for the included studies ranged from 79 to 1,142 patients. Six studies [12–17] adjusted their measure for at least age, gender, Performance Status index, alcohol consumption, education, tumor stages, surgery history, nonsteroidal anti-inflammatory drugs (NSAIDs), or body mass index (BMI). Six articles provided a definition of smoking [11–14, 16, 17]. Three studies analyzed the relationship between smoking intensity and prognosis [12, 14, 16]. Two of them provided HR values of mortality rate [12, 16]. All studies gathered smoking status at the time of diagnosis. The main characteristics of the included studies are summarized in Table 1.

### 3.2. Systematic Review and Meta-Analysis

As we mentioned above, eight studies were eligible for the systematic review and meta-analysis (Table 3). In view of few survival data that had been found directly comparing with current smoker and former smoker, we divided the studies into three groups: current versus never smokers, former versus never smokers, and ever versus never smokers (Table 2). However, only two of the eight matched studies have contained an analysis of the survival of esophageal adenocarcinoma patients [11, 17]: one in both current versus never smoker and former versus never smoker groups (HR = 1.0, 95% CI 0.6–1.7, and HR = 0.9, 95% CI 0.6–1.4, resp.) [17] and the other one in the ever versus never smoker group (HR = 0.86, 95% CI 0.64–1.16) [11]. No significantly increased risk of death was found in those two articles. With the lack of enough data on the survival of EA patients, the following results mainly aimed at the ESCC patients.

### 3.3. Current versus Never Smokers

**Figure 2:**
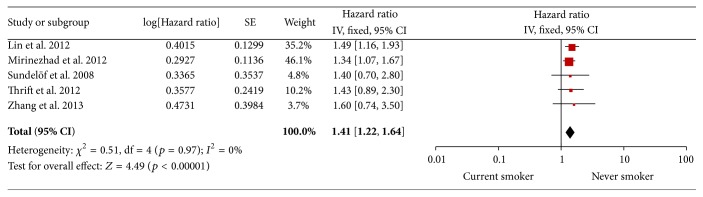
Current smoker versus never smoker.

**Figure 3:**
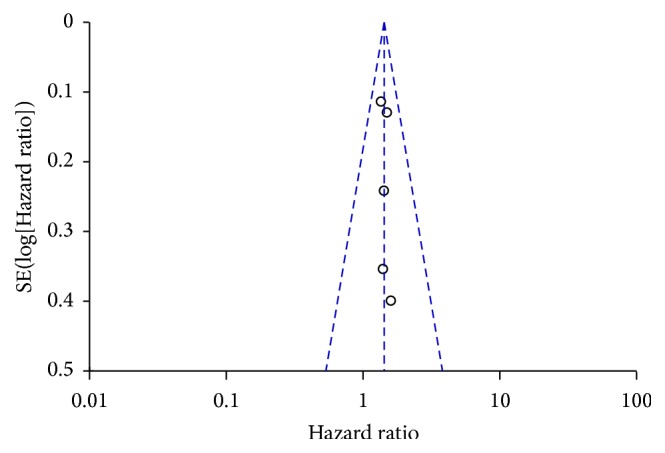
The funnel plot of current versus never smoker.

All five trials analyzed the survival of current and never smokers [10, 13, 15–17]. The *I* ^2^ was 0% indicating that there was no heterogeneity in the pooled studies. Thus, in the fixed-effects model, current smokers showed a 41% higher mortality than never smokers (HR = 1.41, 95% CI 1.22–1.64, *p* < 0.00001, *I* ^2^ = 0%). A funnel plot of studies including survival of current and never smokers was created to estimate publication bias. It showed symmetric distribution, indicating that there may be minimal publication bias in the included studies. Begg's test showed no significant publication bias (*p* = 0.501). These analyses enhanced the reliability of our meta-analysis. See Figures 2 and 3.

**Figure 4:**
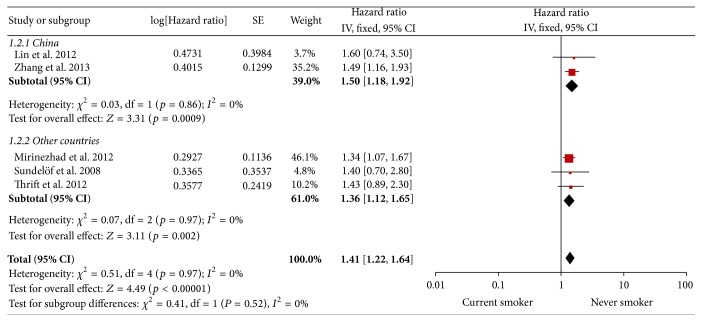
Subgroup analysis of current versus never smoker.

In subgroup analysis, the adverse effects of current smoking on prognosis have been shown in China and other countries (HR = 1.5, 95% CI 1.18–1.92, and HR = 1.36, 95% CI 1.12–1.65, resp.). See Figure 4.

### 3.4. Former versus Never Smokers

**Figure 5:**
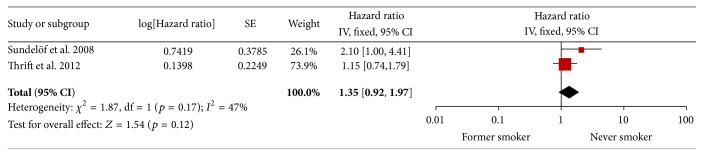
Former versus never smoker.

Two studies reported the survival of former and never smokers [16, 17]. The result of the test for heterogeneity was also not significant (*p* = 0.42). The pooled estimate showed a HR of 1.35 (95% CI 0.92–1.97, *p* = 0.17, *I* ^2^ = 47%) in favor of patients who quit smoking. See Figure 5.

### 3.5. Ever versus Never Smokers

**Figure 6:**
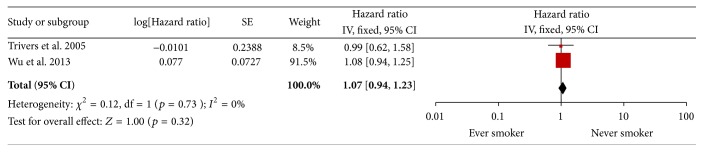
Ever versus never smoker.

Two studies combined groups of current and former smokers in a category of ever smokers [11, 14]. However, no significant difference was found in survival between ever and never smokers in ESCC (HR = 1.07, 95% CI 0.94–1.23, *p* = 0.32, *I* ^2^ = 0%). See Figure 6.

### 3.6. Smoking Intensity

Three studies reported the relationship between survival and smoking intensity (Table 4) [12, 14, 16]. Two of them assessed intensity by smoking pack years (PY) and one by the cigarettes per day (C/D). In Shitara et al.'s study [12], 364 EC patients were divided into two groups according to the smoking status: nonheavy smokers (PY < 20) versus heavy smokers (PY *⩾* 20). The 3- and 5-year survival rates were likely lower in the heavy smoking group, especially in patients treated by chemoradiotherapy (HR = 2.43, 95% CI 1.38–4.27, *p* = 0.002). When the researchers further divided heavy smokers into two subgroups according to PY (20 < PY < 40 and PY > 40), the dose-response relationship was suggestive but not statistically significant in the multivariate analyses. No trend of increasing risk in early death was found across consumption categories (*p*-trend = 0.41 and *p* = 0.53) in other two studies. See Table 4.

## 4. Discussion

In clinical practice, we found that nearly 20~30% cancer patients were addicted to cigarettes, and many cancer survivors continue to smoke even after diagnosis [23]. Smoking is a well-known risk factor for esophageal cancer [23–25]. Yet, there are few studies that directly evaluate smoking as a prognostic factor for esophageal cancer [26]. To our knowledge, this is the first quantitative systematic review and meta-analysis on this topic, based on seven case-control studies [10–13, 15–17] and one cohort study [14] involving more than 4,000 cases. Only two of the eight matched studies have contained an analysis of the survival of esophageal adenocarcinoma patients [11, 17]. Due to lack of sufficient data on the survival of EA patients, our results are mainly targeted at the ESCC patients.

An increased risk of 41% and 35% was estimated for current and former smokers compared to never smokers, respectively, even though the latter one was not statistically significant. When stratifying studies according to geographic areas, an adverse effect of current compared with never smoking was observed in China and other countries (Iran, Australia, and Sweden). The pooled HRs of two subgroups are very close (HR = 1.5, 95% CI 1.18–1.92, and HR = 1.36, 95% CI 1.12–1.65, resp.). These findings indicate that geographical difference would not interfere with the reliability of this meta-analysis. Unfortunately, neither Zhang et al.'s [10] nor Mirinezhad et al.'s [15] studies provide a clear definition of smoking status. Since a cigarette smoker may not just be a current smoker, we conduct a sensitivity analysis which excluded these two studies in the current smoking analysis. The conclusions were not altered in sensitivity analyses (HR = 1.47, 95% CI 1.19–1.82, *p* = 0.0004).

We fail to replicate the similar result in the ever smoker group (HR = 1.07, 95% CI 0.94–1.23). It may be due to a different definition of smoking status. For example, Sundelöf et al.'s study [17] defined former smoking as having quit smoking for at least two years and ever smoking as individuals smoking regularly at least one cigarette per day or at least one cigar or pipe per week during a period of at least six months, while in Shitara et al.'s study [12] former smokers were defined as those who quit smoking at least 1 year before the survey.

Looking at smoking intensity, we found significantly worse survival rates in esophageal squamous-cell cancer patients with a history of heavy tobacco smoking (PY of more than 15 or 20). But, in Shitara et al.'s study, the dose-response relationship was not statistically significant when they divided heavy smokers into two groups according to PY (20 < PY < 40 and PY > 40). Similar result can be found in Thrift et al.'s study [16], when PY of more than 15 was defined as heavy smoking. The difference between experimental and clinical observation findings may be attributed to the following reasons. Firstly, no consensus on the threshold of heavy smoking has been reached around the world. Secondly, all the collected studies gathered smoking history prior to the diagnosis and did not evaluate the impact of behavioral change in smoking after diagnosis, which might also be an interfering factor. To our knowledge, a substantial number of studies have applied this value (PY 20 or Brinkman index of 400) as the threshold of heavy smoking [12, 27–29]. Also, in NCCN Guideline, individuals (age *⩾* 50 y) with more than 20 PY history of smoking tobacco are selected as high risk group for lung cancer [30]. Due to the lack of consensus on the threshold of heavy smoking, Shitara et al.'s [12] point might be more reasonable.

Overall, our review suggests that smoking prevention and cessation would be beneficial for prolonging EC patients' survival. ASCO Tobacco Cessation Guideline published in 2015 (version 1) also recommend that patients with cancer who continue to use tobacco have poorer treatment outcomes compared to their counterparts who do not use tobacco, regardless of whether the cancer was tobacco related [31].

Possible mechanisms linking tobacco exposure and EC outcomes are not yet completely clear. Taghavi et al. [32] found that overexpression of p53 in association with cigarette smoking may play a critical role in ESCC. In addition, p21 overexpression was found to be associated with poor prognosis, specifically in the operable ESCC patients. Moreover, Yamashita et al. [33] revealed that smoking might vary the activity of the 5-FU-related metabolic enzymes, resulting in poor curative effect. 5-Fluorouracil (5-FU) is an anticancer agent widely used in the treatment of digestive tract tumors. Yang et al. [34] showed that smoking can also cause tumor hypoxia, affecting its sensitivity to chemotherapy. So, we speculate that smoking not only is inducing malignant transformation of normal cells but may also change tumor cell gene or related metabolic activity and thus make tumor cells more aggressive and have poorer sensitivity to radiotherapy and chemotherapy. Furthermore, given that smoking causes other complications, part of the excess risk of smoking is that EC patients may succumb to cardiovascular or respiratory disease. Thrift et al. [16] indicated that effects on EC-specific mortality might be driven by some certain characteristics, such as age, sex, pretreatment AJCC tumor stage, treatment, and presence of comorbidities (HR = 1.34, 95% CI 1.02–1.75).

Our study points to areas of research that need more attention to enable a more complete understanding of the role in EC survival and the potential to enhance survival of EC patients by smoking prevention and cessation.

This review and meta-analysis has limitations. First, neither Zhang et al.'s [10] nor Mirinezhad et al.'s [15] studies provide a clear definition of smoking status. A cigarette smoker may not just be a current smoker. Second, based on the predetermined search strategy, we cannot rule out the possibility of having missed relevant articles, especially when the articles are written in languages other than English. Third, no enough information was found on the relationship between smoking and the survival of esophageal junction cancer or esophageal adenocarcinoma; our conclusions mainly focus on the prognosis of ESCC patients. Fourth, meaningful meta-analyses could only be carried out on the increased mortality for current and former smokers compared with never smokers. Comparability was interfered by various definitions and categorizations of smoking exposure, heterogeneity in inclusion and exclusion criteria, and covariates adjusted for [35]. Fifth, we combined results from cohort studies and case-control studies together in the meta-analysis. Strictly speaking, it is not very appropriate. But there are too few articles included in the ever versus never smokers group analysis. Similar statistical methods could be found in an article published in Journal of Clinical Oncology [36]. Moreover, worried about taking blame, some EC patients may refuse to provide real smoking history when visiting their physicians. Finally, there may have been negative studies that were never published, even though there was no indication of publication bias in our meta-analyses.

## 5. Conclusions

In summary, our pooled results support the existence of harmful effects of smoking on survival even after esophagus cancer diagnosis. Tobacco control and smoking cessation should be considered as an important part of a long-term treatment of esophagus cancer. Large population-based and well-designed studies are needed to further clarify the benefit of smoking prevention and cessation for EC patients.

## Figures and Tables

**Table 1:** Baseline characteristics of all included studies.

Study	Country	Year	Type of study	Recruitment	Follow-up		Subjects	Gender	Age		Stage	Definition of smoking	Adjustment	Histology
					End	Median			Range	Median				
Zhang et al. [10]	China	2013	Case control	2009~2010	Dead or 2012/12/31	NR	79	F = 10 M = 69	38~84	63	I~IV	NR	NR	ESCC
Trivers et al. [11]	USA	2005	Case control	1993~1995	2000/10/28	NR	1142	F = 255 M = 887	30~79	NR	I~IV	Y	NR	EA/ESCC/OGA 293/221/367
Shitara et al. [12]	Japan	2010	Case control	2001~2005	NR	5.6 y (2.1–7.9)	363	F = 50 M = 313	33~84	62	I~IV	Y	a, b, c, d, e, f, g	ESCC
Lin et al. [13]	China	2012	Case control	1990~2005	NR	6.5 y (1–20)	643	F = 189 M = 454	NR	NR	IA~IIA	Y	a, b, d, g, k, m	ESCC
Wu et al. [14]	Taiwan, China	2013	Cohort	2000~2008	2008/12/31	NR	718	F = 44 M = 674	NR	59.8 ± 11.6	I~IV	Y	a, b, d, g, h	ESCC
Mirinezhad et al. [15]	Iran	2012	Case control	2006~2011	NR	NR	460	F = 205 M = 255	20~90	65.8 ± 12.2	II~IV	NR	a, b, i, h, j, k, e, g, l	EA/ESCC 26/434
Thrift et al. [16]	Australia	2012	Case control	2001~2005	NR	6.4 y	301	F = 129 M = 172	18~79	66	I~IV	Y	a, b, g, m, n	ESCC
Sundelöf et al. [17]	Sweden	2008	Case control	1995~1997	2004/12/31	NR	580	F = 104 M = 476	NR	NR	NR	Y	a, b, p, d, q	EA/ESCC/GCA 177/159/244

NR: not reported; Y: have a clear definition of smoking status; F: female; M: male; a: age; b: gender; c: performance status index; d: alcohol consumption; e: histology; f: tumor length; g: UICC stage; h: education; i: residence; j: tumor site; k: surgery history; l: tumor differentiation; m: treatment; n: complication; o: do sports; p: gastroesophageal reflux disease (GERD); ESCC: esophageal squamous-cell carcinoma; EA: esophageal adenocarcinoma; OGA: other (noncardia) gastric adenocarcinomas; GCA: gastric cardia adenocarcinoma.

**Table 2:** Other important characteristics of included studies.

Comparison	Study	Median survival (month)	1-year survival rate	3-year survival rate	5-year survival rate	ESCC HR, 95% CI for OS	EA HR, 95% CI for OS
Current versus never smokers	Zhang et al. [10]	NR	NR	NR	NR	1.605 (0.736–3.497)	NR
	Lin et al. [13]	NR	NR	54% versus 67%	46% versus 64%	1.494 (1.157–1.928)	NR
	Mirinezhad et al. [15]	14.47 versus 13.83	61% versus 56%	21% versus 28%	21% versus 21%	1.34 (1.08–1.69)	NR
	Thrift et al. [16]	NR	66% versus 76%	NR	22% versus 48%	1.42 (0.89–2.28)	NR
	Sundelöf et al. [17]	NR	NR	NR	NR	1.4 (0.7–2.8)	1.0 (0.6–1.7)

Former^a^ versus never smokers	Thrift et al. [16]	NR	63% versus 76%	NR	28% versus 48%	1.15 (0.74–1.79)	NR
	Sundelöf et al. [17]	NR	NR	NR	NR	2.1 (1.0–4.4)	0.9 (0.6–1.4)

Ever^b^ versus never smokers	Trivers et al. [11]	NR	NR	NR	NR	0.99 (0.62–1.59)	0.86 (0.64–1.16)
	Wu et al. [14]	10 versus 12	NR	NR	NR	1.08 ( 0.82–1.43)	NR

^a^Used to smoke, but now quit smoking; ^b^including current and former smokers; NR: not reported.

**Table 3:** Results of quality assessment by Newcastle-Ottawa Scale.

Study	1	2	3	4	5A	5B	6	7	8	Scores
Case control										
Zhang et al. [10]	☆	☆	☆	☆	—	—	☆	☆	☆	7
Shitara et al. [12]	☆	☆	☆	☆	☆	☆	☆	☆	—	8
Lin et al. [13]	☆	☆	☆	☆	☆	☆	☆	☆	—	8
Mirinezhad et al. [15]	☆	☆	☆	☆	—	—	☆	☆	☆	7
Thrift et al. [16]	☆	—	☆	☆	☆	☆	☆	☆	☆	8
Sundelöf et al. [17]	☆	☆	☆	☆	☆	☆	☆	☆	—	8
Trivers et al. [11]	☆	☆	☆	☆	☆	☆	☆	—	—	7
Cohort										
Wu et al. [14]	☆	☆	☆	☆	—	☆	☆	☆	☆	8

For case-control studies, 1 indicates adequate definition of cases, 2 cases are representative of population, 3 community controls, 4 controls have no history of smoking, 5A study controls for age and gender, 5B study controls for additional factor(s), 6 ascertainment of exposure by blinded interview or record, 7 the same method of ascertainment used for cases and controls, and 8 nonresponse rate the same for cases and controls. For cohort studies, 1 indicates exposed cohort truly representative, 2 nonexposed cohort drawn from the same community, 3 ascertainment of exposure, 4 outcome of interest not present at start, 5A cohorts comparable on basis of age and gender, 5B cohorts comparable on other factor(s), 6 quality of outcome assessment, 7 follow-up long enough for outcomes to occur (at least 1 year), and 8 complete accounting for cohorts (75% follow-up or description provided of those lost). Newcastle-Ottawa Scale (NOS): see: http://www.ohri.ca/programs/clinical_epidemiology/oxford.asp.

**Table 4:** Mortality and survival of EC patients for different studies.

Smoking intensity (pack year PY or cigarette/day C/D)	Author	Mean survival time (month)	Mortality rate	
			HR	95% CI
PY < 20	Shitara et al. [12]		1	—
20 ⩽ PY			1.73	(1.12–2.68)
20 ⩽ PY < 40			1.77	(1.09–2.89)
40 ⩽ PY			1.69	(1.06–2.67)

Never smoked	Thrift et al. [16]		1	—
0 < PY < 15			1.44	(0.89–2.31)
15 ⩽ PY < 30			0.99	(0.59–1.65)
30 ⩽ PY			1.26	(0.79–2.02)

20 *⩾* C/D	Wu et al. [14]	20.6 ± 27.0		
C/D > 20		19.1 ± 25.6 *p* = 0.03		

PY: pack years; C/D: cigarettes per day.
